# Mapping Tooth Loss at the Tooth Level: Associations with Demographic, Health, and Behavioral Factors

**DOI:** 10.1177/23800844251384952

**Published:** 2025-10-22

**Authors:** K.M. McCormick, G. Mejia, L. Luzzi, L. Jamieson

**Affiliations:** 1 Australian Centre for Population Oral Health, The University of Adelaide, Adelaide, South Australia; 2 The University of North Carolina at Chapel Hill

**Keywords:** tooth loss, mouth, edentulous, periodontitis, socioeconomic factors, diabetes mellitus, smoking

## Abstract

**Objective:**

This cross-sectional observational study analyzed patterns of tooth loss across different population groups using National Health and Nutrition Examination Survey (NHANES) data from 2009 to 2018, aiming to identify the teeth most at risk and the key contributing factors.

**Methods:**

Data from 24,945 adults aged 20 y and older were analyzed. Tooth presence/absence was modeled in relation to age, income, education, smoking history, and glycemic status. To improve robustness, missing covariate data were imputed using k-nearest neighbors, and survey weights were applied in regression analyses to account for NHANES’s complex sampling design. A 2-step analytic approach was used: (1) survey-weighted quasi-Poisson regression estimated associations with total tooth count and (2) unweighted generalized estimating equations identified explanatory variables associated with individual tooth loss, accounting for intraoral clustering. Dental implants were excluded.

**Results:**

Tooth-level models suggested distinct intraoral and demographic patterns. Males retained more posterior teeth but lost more mandibular incisors than females did. Older adults (≥60 y) exhibited markedly higher loss in mandibular anterior and premolar regions. High-income and high-education participants retained more teeth overall but were more likely to lack first premolars, likely reflecting orthodontic extractions. Smoking and diabetes were associated with anterior tooth loss, especially in visible, functionally important regions. Glycemic control showed a clear gradient, with low-normal HbA1c levels protective in posterior sites.

**Conclusions:**

Tooth-level modeling suggested anatomically and socially patterned vulnerabilities often masked in whole-mouth analyses. Findings underscore the need for site-specific prevention strategies that address biological risk and structural inequity, advancing more equitable approaches to oral health care and policy.

**Knowledge Transfer Statement:**

This study identified which specific teeth are most at risk of loss across age, health, and socioeconomic groups. Understanding these tooth-level patterns can help clinicians tailor preventive care to vulnerable patients, guide patient education, and inform public health strategies that target oral health disparities. These findings support more precise, equitable dental care to help people keep their natural teeth longer.

## Introduction

Tooth loss is more than a clinical endpoint: it is a visible marker of cumulative oral and systemic burden, closely tied to quality of life and social participation (Gerritsen et al. 2010; Papadaki and Anastassiadou 2012; Peres et al. 2019). When teeth are lost, especially in functionally or esthetically critical regions, individuals may experience compromised nutrition, reduced confidence, and heightened vulnerability to psychological distress (Hao et al. 2024). As noted in global health frameworks, patterns of tooth loss often mirror broader social and health inequities, particularly where access to preventive care is limited (World Health Organization [WHO] 2024). Its association with chronic conditions—including cardiovascular disease, type 2 diabetes, and cognitive decline—further underscores its relevance as a whole-body health indicator (Alvarenga et al. 2019; Ahmadinia et al. 2022; Matsuyama et al. 2023). In practical terms, the loss of natural teeth diminishes masticatory function and speech clarity, with implications for daily living and long-term well-being (Kaurani et al. 2024; Parisius et al. 2024).

Age and socioeconomic status (SES) are key determinants of oral health disparities, influencing access to preventive care and treatment-oriented care. Older adults face elevated tooth loss risk due to cumulative exposure to dental caries, restoration failure, trauma, and periodontal disease (WHO 2024). Age-related frailty, reduced immune function (Preshaw et al. 2017), and diminished salivary flow (Toan and Ahn 2021) further exacerbate these risks. SES influences oral health through both material barriers and psychosocial pathways (Sanders et al. 2006). Low household income restricts access to care, increasing the risk of untreated disease and tooth loss (Singh et al. 2019). Educational attainment shapes health behaviors, beliefs about oral care, and service use (Moffitt et al. 2011), while limited education is linked to reduced awareness and access (Centers for Disease Control and Prevention [CDC]).

Behavioral and systemic health factors, particularly smoking and diabetes, are well-established contributors to tooth loss. Smoking impairs periodontal health by reducing gingival blood flow, weakening immune responses, and increasing susceptibility to infection, all of which undermine the body’s ability to recover from oral inflammation (Hanioka et al. 2011). Independently, diabetes promotes systemic inflammation and delays wound healing, creating conditions that accelerate periodontal breakdown and increase the risk of tooth loss even in the absence of other risk factors (Barman and Koh 2020).

Previous research on tooth loss has focused on aggregate analyses, such as whole-mouth or quadrant-based assessments. While informative, these approaches may obscure tooth-specific patterns of vulnerability that could inform more targeted interventions. By examining individual teeth, it may be possible to identify how demographic, behavioral, and systemic factors influence disease risk and tooth loss patterns at a more granular level. For instance, it is unclear whether increased risk of tooth loss associated with SES affects all teeth equally or whether certain teeth are more vulnerable. SES-related tooth loss may be concentrated in specific teeth, highlighting localized vulnerabilities that could inform targeted interventions to reduce disparities in oral health outcomes. To build on this, tooth-specific analyses may uncover patterns of heightened vulnerability in particular teeth or regions of the mouth associated with sex, age, education, income disparities, glycemic status, and smoking history. To address this, our study leverages data from the National Health and Nutrition Examination Survey (NHANES) in the United States, a nationally representative dataset that includes detailed information on individual tooth presence, demographic factors, and health behaviors. Specifically, we aim to answer the following research questions:

1. Which individual teeth are at the highest risk of loss across distinct levels of sex, age, household income, education attainment, smoking history, and glycemic status?
2. To what extent do these associations persist after controlling for the other variables?

By focusing on individual teeth, our study aims to generate actionable insights into localized tooth loss patterns, forming a foundation for tailored prevention strategies that address oral health disparities.

## Method

### Data

This cross-sectional observational study used data from the National Health and Nutrition Examination Survey (NHANES) 2009–2018 cycles. NHANES is a nationally representative survey conducted by the National Center for Health Statistics (NCHS), combining interviews, physical examinations, and laboratory tests. Written informed consent was obtained from all participants, and study procedures were approved by the NCHS Research Ethics Review Board.

### Sample and Variables

Participants aged 20 y or older who completed the demographic and smoking questionnaires, underwent a dental examination, and had glycohemoglobin (HbA1c) measured were included. The final analytic sample comprised 24,945 participants (mean age = 50.3 y, standard deviation [SD] = 17.3 y; 51.1% female). Tooth-specific loss was assessed from dental examination data, with each tooth coded as present (1) or absent (0). Teeth recorded as “not present” (19.2%), “permanent root fragment present” (1.1%), or “implant” (0.2%) were classified as absent.^1^ NHANES tooth codes were converted to the Fédération Dentaire Internationale (FDI) 2-digit system (ISO 3950) to ensure anatomical consistency with international research standards. To account for potential variation across data collection periods, participants were grouped by NHANES survey cycle.

Demographic variables included age. categorized as 20 to 34, 35 to 59, or 60+ y, and sex, coded as male or female. Socioeconomic variables included household income, grouped as low (<$35,000), medium ($35,000–$99,999), or high (≥$100,000), based on Pew Research Center and U.S. median income thresholds (NCES 2011; Kochhar et al. 2015;). Educational attainment was categorized as low (less than high school), medium (high school graduate, general educational development [GED] certificate, some college, or associate’s degree), and high (college degree or higher).

Health-related variables included smoking history, categorized as having “smoked at least 100 cigarettes in one’s lifetime” (ever-smoker) or not (never-smoker),^2^ and glycemic status (based on HbA1c levels) were categorized as low-normal (<5.4%), high-normal (5.4–5.6%),^3^ prediabetes (5.7%–6.4%), and diabetes (≥6.5%).^4^

### Missing Data and Imputation

Four variables contained missing data: income (9.9%), glycemic status (4.2%), education (0.2%), and smoking history (0.06%). To minimize bias and preserve statistical power, imputation was performed prior to deriving categorical variables for age, education, income, and glycemic status. Responses of “refused” and “don’t know” were recoded as missing, along with the ambiguous “>$20,000” income category, which overlapped the predefined medium- and high-income categories, preventing reliable assignment without introducing misclassification bias. Dentition variables were excluded from the imputation model to avoid circularity with the outcome. We implemented multiple imputation by chained equations (MICE) using predictive mean matching, a semi-parametric method suitable for mixed data types and robust to nonnormality. This approach preserves the multivariate structure of the data while reducing bias from missing-at-random and potential missing-not-at-random mechanisms, particularly relevant for variables such as income. Five imputed datasets (m = 5) were generated in line with standard practice, which balances statistical efficiency and computational burden (Rubin 1996).

### Statistical Analysis

A 3-step analytic approach was used to examine factors associated with tooth loss: (1) survey-weighted descriptive statistics were calculated for total dentition count, (2) population-level associations were modeled using negative binomial regression, and (3) intraoral patterns were assessed using unweighted tooth-level models.

### Descriptive and Heatmap Analysis

Weighted descriptive statistics were used to estimate population-level summaries (i.e., mean and standard deviation) of dentition counts, stratified by key explanatory variables. In contrast, intraoral heatmaps were constructed using unweighted data to visualize anatomical patterns of tooth loss across the dentition. We deliberately omitted survey weights from the heatmaps to preserve interpretability at the individual tooth level, where weighting can obscure localized variation (Gelman 2007). These visualizations were stratified by age, education, income, glycemic status, and smoking history and were intended to highlight tooth-specific vulnerabilities within subgroups rather than to estimate population prevalence.

### Weighted Whole-Mouth Regression

Survey weights were applied to account for NHANES’s complex multistage sampling design, including stratification, clustering, and unequal selection probabilities. We adjusted the 2-y sampling weights by dividing them by the number of combined cycles (*N* = 5) to ensure correct estimation across the 10-y pooled sample. A survey-weighted quasi-Poisson regression model was used to obtain robust estimates of incidence rate ratios (IRRs) with 95% confidence intervals for the number of missing teeth (range: 0–28), addressing overdispersion in count data. This approach follows best-practice recommendations for NHANES analysis (Korn and Graubard 1991) and supports valid population-level inference by mitigating bias associated with unweighted models. Explanatory variables included age group, sex, smoking history, glycemic status, education level, income level, and NHANES cycle. Reference groups were defined as female (sex), age 35 to 59 y (age), medium education, medium household income, never-smoker, high-normal glycemic status (5.4%–5.6% HbA1c), and NHANES cycle F (2009–2010).

### Unweighted Tooth-Level Regression

To examine intraoral patterns of tooth loss, we fitted separate unweighted generalized estimating equation (GEE) models for each of the 28 teeth, using a Poisson distribution with a log link and accounting for clustering at the individual level. Each model included the same covariates and reference groups as the whole-mouth analysis. Models were deliberately left unweighted to preserve the anatomical interpretability of tooth-specific variation rather than to generate population-level estimates (Gelman 2007). Estimates (B) are unstandardized log coefficients from unweighted Poisson GEE models. Prevalence ratios are not reported, as the models were unweighted and thus not directly generalizable to the U.S. population. Coefficients were plotted by tooth position to visualize spatial gradients in risk across subgroups. An exchangeable working correlation structure was selected a priori to account for within-person clustering. To aid interpretability and conserve space, only coefficients with 95% confidence intervals that excluded zero are reported in text, unless otherwise specified.

### Statistical Software

All analyses were conducted using R (version 4.3.1; R Core Team 2023) in RStudio. Survey weights were applied using the survey package (Lumley 2023), which accounts for the complex multistage sampling design of NHANES, including stratification, clustering, and adjustment of 2-y weights across combined cycles. Weighted descriptive statistics were generated using the srvyr package (Ellis and Schneider 2023), and missing sociodemographic data were imputed using MICE with predictive mean matching (m = 5 datasets) via the mice package (Van Buuren and Groothuis-Oudshoorn 2011). GEEs were fitted using the geepack package (Halekoh et al. 2006), allowing for clustering of observations within individuals.

This study adhered to the STROBE (Strengthening the Reporting of Observational Studies in Epidemiology) guidelines for observational research.

## Results

### Descriptive and Heatmap Analysis

Sample distributions and dentition rates are summarized in Table 1. Weighted analysis suggests that, on average, participants had nearly 5 missing teeth (mean = 4.8, SD = 0.1), although this masked substantial variation across the population. Age was a particularly strong driver: older adults (60+ years) had, on average, nearly 10 missing teeth (mean = 9.7, SE = 0.2), while younger adults (20–34 years) averaged just 1 (mean = 1.1, SD = 0.1). Sex differences were modest but evident. Females had slightly more missing teeth than males did (mean = 4.9 vs. 4.7; SD = 0.1 and 0.2, respectively), although this gap was relatively small in practical terms. Interestingly, variation across survey cycles was minor. Despite spanning a decade of data collection, average tooth loss ranged only slightly across NHANES cohorts (mean = 4.4 to 5.4), suggesting relative temporal stability in population-level tooth loss.

**Table 1.** Descriptive Statistics.

Characteristic	Sample Size, *n* (%)	Weighted Missing Teeth (Mean ± SD)
Total	24,495 (100)	4.8 ± 0.1
Cohort		
F (2009–2010)	4,126 (16.8)	5.4 ± 0.3
G (2011–2012)	4,911 (20.0)	4.7 ± 0.3
H (2013–2014)	5,202 (21.2)	5.0 ± 0.4
I (2015–2016)	5,199 (21.2)	4.4 ± 0.3
J (2017–2018)	5,057 (20.6)	4.7 ± 0.2
Sex		
Male	11,973 (48.9)	4.7 ± 0.2
Female	12,522 (51.1)	4.9 ± 0.1
Age (y)		
20–34	5,626 (23.0)	1.1 ± 0.1
35–59	10,482 (42.8)	4.0 ± 0.1
60+	8,387 (34.2)	9.7 ± 0.2
Smoking history		
Never-smoker (<100 cigarettes)	13,858 (56.6)	3.3 ± 0.1
Ever-smoker (≥100 cigarettes)	10,637 (43.4)	6.7 ± 0.2
Glycemic status		
Low normal <5.4%	7,745 (31.6)	2.7 ± 0.1
High normal 5.4–5.6%	6,491 (26.5)	4.4 ± 0.1
Prediabetes 5.7–6.4%	7,259 (29.6)	7.0 ± 0.2
Diabetes ≥6.5%	3,000 (12.2)	9.1 ± 0.3
Household income		
Low (<$35,000 p/a)	10,172 (41.5)	7.9 ± 0.2
Medium ($35–99k p/a)	6,966 (28.4)	4.7 ± 0.2
High (≥$100k p/a)	7,357 (30.0)	2.6 ± 0.1
Educational attainment		
Low	5,664 (23.1)	8.8 ± 0.3
Medium	12,819 (52.3)	5.2 ± 0.1
High	6,012 (24.5)	2.2 ± 0.1

k = thousand; p/a = per annum; SD = standard deviation.

Dentition represents the mean number of retained natural teeth. Educational achievement is categorized as “low” (no formal education, some primary or secondary education without a diploma), “medium” (high school or General Educational Development diploma, some postsecondary education without a degree, associate’s degree, or apprenticeship), and “high” (bachelor’s degree or higher).

Tooth loss followed a striking social gradient. Those in the low-income group had, on average, more than 3 times the number of missing teeth as those in the high-income group did (mean = 7.6 vs. 2.7; SD = 0.2 and 0.1). Education showed a similar pattern: participants with low educational attainment had, on average, more than 4 times the number of missing teeth compared with those with high education (mean = 8.7 vs. 2.2; SD = 0.3 and 0.1).

Patterns by health status were also stark. Participants living with diabetes had the highest levels of tooth loss (mean = 8.9, SD = 0.3), while those with low-normal HbA1c had the lowest (mean = 2.7, SD = 0.1). Smoking history showed a clear divide: ever-smokers had, on average, double the number of missing teeth compared with never-smokers (mean = 6.7 vs. 3.3; SD = 0.2 and 0.1).

Patterns of tooth loss varied markedly by anatomical location and demographic subgroup, as illustrated in the unweighted heatmaps (Fig. 1). Across all strata, posterior teeth—particularly the mandibular molars (teeth 36, 37, 46, 47)—exhibited consistently higher rates of absence than anterior teeth did. A clear gradient of increasing loss was observed among older participants and those with lower income or poorer glycemic control. In contrast, anterior teeth, especially the mandibular incisors and canines, were more frequently retained, although losses still rose with age and socioeconomic disadvantage. A distinct pattern emerged for the first premolars (teeth 14, 24, 34, 44), which showed elevated rates of absence in younger, healthier, and higher-SES groups—suggestive of orthodontic extraction rather than disease. This pattern was less apparent in older or disadvantaged subgroups, consistent with a transition to pathology-driven tooth loss. These intraoral differences were observed across all stratified heatmaps, underscoring anatomical and socioeconomic gradients in tooth vulnerability.

**Figure 1.**
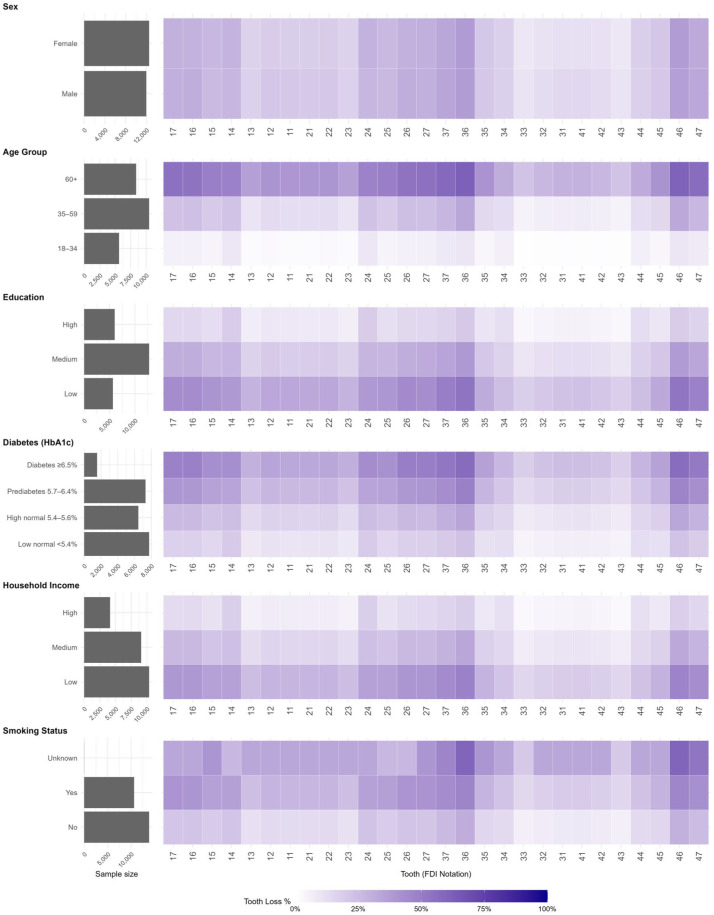
Unweighted heatmaps showing the prevalence of tooth loss by tooth number across sociodemographic groups. Each tile represents a tooth (FDI notation), colored by the proportion of participants with that tooth missing within each subgroup. Stratification is shown by key variables including age, sex, income, education, glycemic status, and smoking history, with sample sizes indicated for each group. Deeper colors represent a higher prevalence of tooth loss. Patterns reveal consistent anterior–posterior gradients, with marked disparities observed in the mandibular anterior and premolar regions among lower socioeconomic and clinical risk groups. Full descriptions of variable categories are provided in Table 1.

### Whole-Mouth Analysis (Weighted)

Weighted adjusted Poisson regression results, adjusted for all covariates in the model, are presented in Table 2. Several demographic, health, and socioeconomic indicators were significantly associated with tooth loss. Age was the most influential explanatory variable. Compared with adults aged 35 to 59 years (reference), younger adults aged 20 to 34 y had markedly fewer missing teeth (IRR = 0.28), while older adults aged 60+ y had substantially more (IRR = 2.09; 95% confidence interval [CI]: 1.99–2.19). Males had slightly fewer missing teeth than females did (IRR = 0.95; 95% CI: 0.92–0.99).

**Table 2.** Whole-Mouth Survey-Weighted Quasi-Poisson Regression Model.

Characteristic	IRR (95% CI)	*P* Value
Intercept	3.28 (3.00, 3.59)	<.001
Cycle		
F (2009–2010)	Ref	NA
G (2011–2012)	0.99 (0.90–1.08)	.812
H (2013–2014)	1.11 (0.97–1.26)	.125
I (2015–2016)	0.95 (0.87–1.04)	.296
J (2017–2018)	1.02 (0.93–1.13)	.642
Sex		
Female	Ref	NA
Male	0.95 (0.92–0.99)	.027
Age (y)		
18–34	0.28 (0.26–0.31)	<.001
35–59	Ref	NA
60+	2.09 (1.99–2.19)	<.001
Smoking history		
Never-smoker	Ref	NA
Ever-smoker	1.57 (1.49–1.64)	<.001
Glycemic status		
Low normal <5.4%	0.92 (0.85–0.98)	.020
High normal 5.4–5.6%	Ref	NA
Prediabetes 5.7–6.4%	1.10 (1.05–1.16)	<.001
Diabetes ≥6.5%	1.28 (1.20–1.37)	<.001
Household income		
Low (<$35,000 p/a)	1.39 (1.32–1.46)	<.001
Medium ($35k–$99k p/a)	Ref	NA
High (> $100,000 p/a)	0.78 (0.73–0.84)	<.001
Educational attainment		
Low	1.33 (1.27–1.39)	<.001
Medium	Ref	NA
High	0.54 (0.50–0.58)	<.001

CI, confidence interval; IRR, incidence rate ratio; k, thousand; NA, not applicable; p/a, per annum; *SD*, standard deviation.

The IRRs are reported relative to the reference group, indicated as “Ref,” and are accompanied by 95% CIs. A 95% CI that includes 1.00 indicates no significant difference (*P* values are reported for completeness). NA indicates values not applicable, as these categories serve as the reference groups for comparisons in the regression analysis. Full descriptions of variable levels are given in Table 1.

Socioeconomic disparities were pronounced. Compared with the medium-income group (reference), individuals in the low-income group had significantly more tooth loss (IRR = 1.39; 95% CI: 1.32–1.46), whereas those in the high-income group had substantially less (IRR = 0.78; 95% CI: 0.73–0.84). A similar pattern was evident for educational attainment: participants with low education had greater tooth loss (IRR = 1.33; 95% CI: 1.27–1.39), while those with high education exhibited markedly lower tooth loss (IRR = 0.54; 95% CI: 0.50–0.58).

Tooth loss was higher among ever-smokers (IRR = 1.57; 95% CI: 1.49–1.64) compared with never-smokers (reference), and a gradient was observed for glycemic status. Relative to participants with high-normal (reference), those with low-normal had significantly fewer missing teeth (IRR = 0.92). Tooth loss increased among those with prediabetes (IRR = 1.10; 95% CI: 1.05–1.16) and was highest among participants with diabetes (IRR = 1.28; 95% CI: 1.20–1.37).

### Tooth-Level Analysis (Unweighted)

Exploratory unweighted tooth-level models indicated potential intraoral differences across demographic groups (Fig. 2; full regression results provided in Appendix Tables S1–S28). Compared with females (reference group), males exhibited significantly lower probabilities of tooth loss at several maxillary and mandibular posterior and premolar sites, particularly on the left side (teeth 13–15, 24–27, 34–36, 44–46; B range = −0.05 to −0.12). In contrast, males were more likely to have lost their mandibular central incisors (teeth 31 and 41; B = 0.09 and 0.10), suggesting a nuanced sex pattern that diverges between anterior and posterior regions.

**Figure 2.**
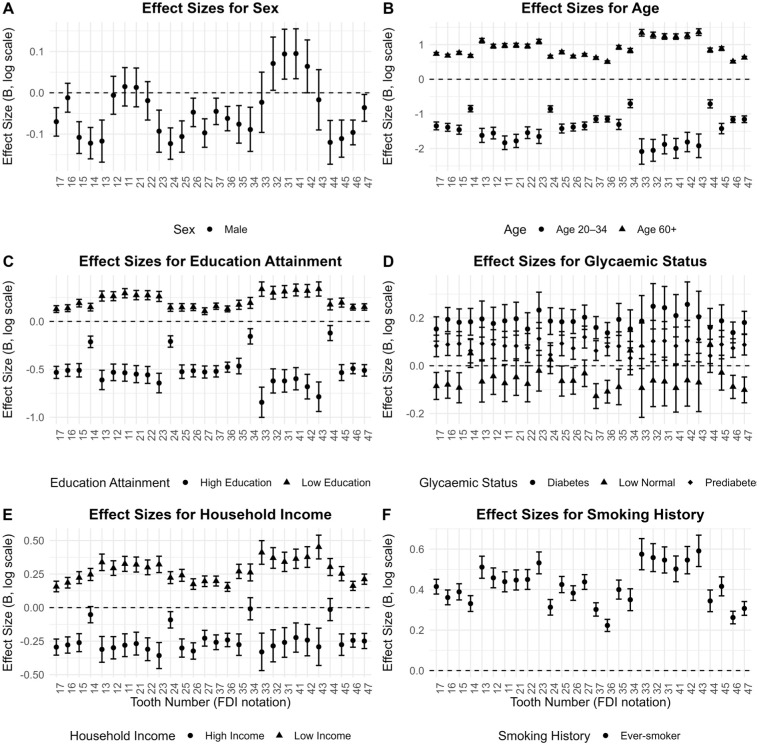
Unweighted, adjusted generalized estimating equation (GEE) model coefficients for explanatory variables of tooth loss across the permanent dentition. Each point represents the log-linked effect size (B) for a given explanatory variable at an individual tooth, controlling for all other covariates in the model. Results are presented relative to the following reference groups: 35 to 59 years (age), female (sex), high-normal (glycemic status), medium income, medium education, and never-smoker (smoking history). Positive coefficients reflect increased probability of tooth loss relative to the reference group; negative coefficients indicate decreased probability. Tooth numbers follow FDI notation and are arranged by arch and quadrant. Visual patterns highlight anterior–posterior gradients and site-specific social and behavioral disparities in retention. Full descriptions of variable levels are given in Table 1.

Age patterns were especially strong. Older adults (60+ y) exhibited a markedly higher association with tooth loss across nearly all teeth, with effects most pronounced in the mandibular anterior regions. For example, lower anterior teeth (31–33, 41–43) showed stronger loss associations (B ranges = 1.23 to 1.36). In contrast, young adults (20–34 y) had a substantially lower probability of tooth loss across the dentition, with the strongest protective effects observed in the mandibular anterior and premolar teeth (B = −1.15 to −2.09). Effects were more muted at the first premolars (14, 24, 34, 44; B = −0.70 to −0.86), where tooth loss may reflect orthodontic extraction rather than disease.

A clear educational gradient was also observed. Compared with those with medium education (reference), participants with low education experienced higher tooth loss associations across most regions, with the most prominent difference at tooth 43 (B = 0.34). In contrast, high education was linked to reduced tooth loss across nearly all sites (B = −0.84 to −0.46), with muted effects at the first premolars, a pattern likely driven by differential access to orthodontic extractions rather than underlying disease.

Glycemic status showed a consistent dose-response gradient. Participants with diabetes (≥6.5% HbA1c) exhibited significantly greater tooth loss across the dentition, especially in the lower anterior region (B = 0.21 to 0.26 for teeth 31–33, 41–43). Participants with prediabetes showed more variable effects, with elevated loss at several teeth (e.g., 27, 23, 31, 43, 42; B = 0.10 to 0.12) compared with those with high-normal glycemic levels. Participants in the low-normal HbA1c group had a significantly lower probability of tooth loss at several posterior sites, particularly molars and second premolars (B = −0.03 to −0.13), underscoring the protective influence of optimal glycemic control.

Smoking effects were widespread and most pronounced in visible, anterior regions. Compared with never-smokers (reference group), ever-smokers had substantially higher tooth loss in the anterior regions (e.g., 11–13, 21–23, 31–33, 41–43; B = 0.44 to 0.59). Although loss was also elevated in posterior teeth (e.g., 36 and 46; B = 0.22 and 0.26), the impact was more muted. These findings highlight the impact of smoking across the lifespan, even at a low-exposure threshold.

Income-related patterns were similarly stark. Compared with the medium-income group (reference), individuals in the low-income group experienced significantly greater loss, especially in the mandibular anterior teeth (e.g., 31–33, 41–43; B = 0.34 to 0.45). In contrast, high-income participants retained more teeth across all regions (B = −0.22 to −0.38), except the first premolars, which were attenuated (e.g., 14, 24, 34, and 44; range B = −0.01 to −0.09), again suggesting the influence of elective extractions for orthodontic purposes for socioeconomic groups.

## Discussion

This study examined tooth loss at an anatomical level of detail not typically captured in population-level research. By modeling loss at the level of individual teeth, we estimated patterns often obscured by the aggregate approaches used in large-scale studies. Mandibular molars were frequently lost across subgroups, but adjusted models suggested they were most at risk among individuals with low education and low income and protective in high-education and high-income groups. Males retained more posterior teeth than females did but were more likely to have lost their mandibular central incisors. This bidirectional pattern reflects distinct trajectories in disease exposure or treatment history and underscores the importance of tooth-level modeling for identifying intraoral manifestations of structural inequity.

A second example emerges in the divergent patterns of first premolar loss. Younger adults from high-income backgrounds were more likely to lack first premolars, likely due to elective orthodontic extractions. In contrast, retention of these teeth in older or low-income participants suggests that loss in these groups is more strongly disease driven. This highlights how social context may shape intraoral patterns of tooth loss and underscores the limitations of whole-mouth summary metrics. A tooth-level perspective estimates region-specific vulnerabilities shaped by social and systemic exposures, supporting more precise clinical risk assessment, nuanced surveillance, and equitable prevention strategies.

The observed vulnerability of anterior teeth, particularly in older and structurally disadvantaged groups, likely reflects the cumulative impact of systemic exposures on biological processes. Subgingival calculus accumulation and chronic inflammation—amplified by smoking and reduced access to care—are well-known contributors to periodontal breakdown in these regions (Thomson et al. 2013; Noor and Al-Bayaty 2015). Similar patterns were reported in the Dunedin study (Thomson et al. 2013), which documented a sharp increase in attachment loss in the mid-30s, especially in the lower incisors—a transition from subclinical disease to irreversible damage.

These findings have practical implications for both public health and clinical care. Tailored health messaging, subsidized preventive services, and integration of oral health into chronic disease management—especially for diabetes and kidney disease—could help prioritize protection of vulnerable teeth. In our models, diabetes was most strongly associated with anterior tooth loss, with weaker effects in posterior regions. This gradient underscores the importance of site-specific monitoring. Preserving natural teeth is vital for maintaining mastication, nutrition, and healthy aging, and untreated oral infections can delay or preclude transplantation (Costantinides et al. 2018). Embedding tooth-specific indicators into surveillance systems and chronic disease pathways could enable earlier identification of periodontal risk and improve equity in care delivery.

Bridging these findings to clinical practice, a site-specific risk profile approach could support more precise treatment planning—targeting both high-risk patients and high-risk teeth for that patient. For example, clinicians might prioritize anterior tooth preservation in patients with diabetes or low-income status or reassess extraction decisions for groups with elevated risk of disease progression. Addressing implicit biases is also critical, as assumptions about a patient’s ability to pay may influence decisions to extract rather than restore teeth. Equitable treatment planning requires recognizing how these dynamics shape access to care and supporting patients across social strata to retain functional, healthy dentition.

### Strengths and Limitations

This study applied a tooth-level exploratory analytical framework to produce a more granular understanding of tooth loss patterns than whole-mouth or quadrant-based approaches. A key strength is the use of NHANES, a large, nationally representative dataset capturing a diverse cross section of the U.S. population. The inclusion of multiple survey cycles (2009–2018) allowed for temporal comparisons, while the use of GEE accounted for clustering of teeth within individuals and produced population-averaged estimates. Missing data were addressed using MICE, incorporating age, sex, education, smoking status, and glycemic status to inform donor selection. This approach helped preserve statistical power and reduce bias under the assumption of data missing at random. However, if unmeasured factors influenced missingness—particularly for income—some residual bias may remain.

Several limitations should be noted. Given the number of comparisons across 28 teeth and multiple explanatory variables, findings should be interpreted as hypothesis generating. The cross-sectional design limits causal inference, and unmeasured variables—such as dietary intake, chronic stress, or genetic predisposition—may have influenced results. Sampling weights were not applied to the tooth-level GEE models, as the focus was on intraoral patterning rather than generating population-level estimates. While this limits generalizability, it enabled more anatomically interpretable comparisons. Future studies could build on these findings using longitudinal designs to confirm site-specific associations, examine how implant placement may reproduce or mitigate structural disparities (particularly in anterior regions), and explore the role of psychosocial exposures such as chronic stress, which may amplify inflammatory responses (Darby 2022) and interact with structural disadvantage to increase periodontal risk (Gottschalk et al. 2020). Longitudinal data will be essential to disentangle these relationships and clarify how demographic, behavioral, and clinical exposures accumulate over time to shape site-specific vulnerability—supporting earlier intervention and more targeted prevention and informing risk-based approaches to clinical decision-making and surveillance design.

## Conclusion

This study highlights the potential value of a tooth-level analytical framework for exploring intraoral patterns of tooth loss that may be masked in whole-mouth analyses. By modeling individual teeth, we observed socially patterned vulnerabilities, such as a tendency toward anterior tooth loss among older adults, males, and low-income individuals and elective premolar extractions among high-income youth. These exploratory findings point to the complexity of tooth loss as both a biological and structural phenomenon and suggest the importance of targeted prevention strategies that account for specific populations, anatomical regions, and life stages. As a hypothesis-generating study, this work provides a foundation for longitudinal research into causal pathways and for developing risk-based surveillance tools using complex survey data. Tooth-level insights may ultimately inform more precise clinical decision-making and more equitable public health strategies to support dentition retention across the lifespan.

## Author Contributions

K.M. McCormick, contributed to conception and design, data acquisition, analysis, and interpretation, drafted and critically revised the manuscript; G. Mejia, contributed to conception, data acquisition and interpretation, critically revised the manuscript; L. Luzzi, L. Jamieson, contributed to interpretation, critically revised the manuscript. All authors have reviewed and approved the final manuscript and agree to be accountable for all aspects of the work.

## Supplemental Material

sj-docx-1-jct-10.1177_23800844251384952 – Supplemental material for Mapping Tooth Loss at the Tooth Level: Associations with Demographic, Health, and Behavioral Factors Supplemental material, sj-docx-1-jct-10.1177_23800844251384952 for Mapping Tooth Loss at the Tooth Level: Associations with Demographic, Health, and Behavioral Factors by K.M. McCormick, G. Mejia, L. Luzzi and L. Jamieson in JDR Clinical & Translational Research

## Notes

1. Dental implants were excluded from this study to focus on the natural dentition.

2. Based on NHANES definitions.

3. We subdivided normoglycemia categories into 2 groups to (1) ensure more balanced group sizes (primary purpose) and (2) explore potential dose-response relationships within this subclinical level (secondary purpose based on the findings of Sezer et al. 2021).

4. As defined by the American Diabetes Association (XXXX) guidelines.
