# Supplementary material for: Mapping Tooth Loss at the Tooth Level: Associations with Demographic, Health, and Behavioral Factors
Source: JDR Clin Trans Res. 2025 Oct 22;11(3):465–75. doi: 10.1177/23800844251384952 (PMC13232582; doi:10.1177/23800844251384952)
Supplement: Supplementary file 1 [file sj-docx-1-jct-10.1177_23800844251384952.docx]

Tooth-level associations between demographic, health, and socioeconomic factors and tooth loss: A cross-sectional analysis of NHANES 2009–2018

Kym M. McCormick^1^, Gloria Mejia^2^, Liana Luzzi^1^ & Lisa Jamieson^1^

^1^Australian Centre for Population Oral Health, The University of Adelaide, Adelaide, South Australia

^2^The University of North Carolina at Chapel Hill

Dr Kym M. McCormick: [kym.mccormick@adelaide.edu.au](mailto:kym.mccormick@adelaide.edu.au)

Dr Gloria Mejia: [gmejia@unc.edu](mailto:gmejia@unc.edu)

Dr Liana Luzzi: [liana.luzzi@adelaide.edu.au](mailto:liana.luzzi@adelaide.edu.au)

Prof Lisa Jamieson: [lisa.jamieson@adelaide.edu.au](mailto:lisa.jamieson@adelaide.edu.au)

Supplementary Materials

Correspondence concerning this article should be addressed to Kym McCormick, Adelaide Dental School, The University of Adelaide, Level 4, 50 Rundle Mall, Rundle Mall Plaza, Adelaide, South Australia 5005. Email: [kym.mccormick@adelaide.edu.au](mailto:kym.mccormick@adelaide.edu.au)

# Tooth-level analysis: GEE models

For clarity of presentation, intercept terms are not reported in the tooth-level regression tables, as they represent the baseline log probability of tooth absence for the reference group and are not clinically interpretable.

## Table S1

Generalized Estimating Equations (GEE) model predicting absence of tooth 17 (reference = presence). Coefficients on log scale (*B*, SE, 95% CI) are shown.

| **Fixed effect** | **Estimate** | **SE** | **95%% CI** | ***p*** |
| --- | --- | --- | --- | --- |
| Age: 20–34 | -1.35 | 0.06 | [-1.46, -1.23] | <.001 |
| Age: 60+ | 0.73 | 0.02 | [0.69, 0.78] | <.001 |
| Education attainment: High | -0.53 | 0.03 | [-0.59, -0.47] | <.001 |
| Education attainment: Low | 0.13 | 0.02 | [0.09, 0.16] | <.001 |
| Glycemic status: ≥6.5% | 0.15 | 0.03 | [0.10, 0.20] | <.001 |
| Glycemic status: Low normal <5.4% | -0.08 | 0.03 | [-0.14, -0.03] | .003 |
| Glycemic status: Prediabetes 5.7–6.4% | 0.08 | 0.02 | [0.04, 0.13] | <.001 |
| Household income: High | -0.29 | 0.03 | [-0.35, -0.23] | <.001 |
| Household income: Low | 0.16 | 0.02 | [0.12, 0.20] | <.001 |
| NHANES cycle: G | 0.04 | 0.03 | [-0.01, 0.10] | .128 |
| NHANES cycle: H | 0.08 | 0.03 | [0.02, 0.13] | .005 |
| NHANES cycle: I | -0.02 | 0.03 | [-0.07, 0.04] | .544 |
| NHANES cycle: J | 0.03 | 0.03 | [-0.02, 0.09] | .212 |
| Sex: Male | -0.07 | 0.02 | [-0.10, -0.04] | <.001 |
| Smoking: Ever-smoker | 0.41 | 0.02 | [0.38, 0.45] | <.001 |

**Note**. Intercept terms are suppressed from tooth-level GEE tables for clarity, as they represent the baseline log probability of tooth absence in the reference group and are not clinically interpretable. Reference groups: Female (sex), age 35–59 years (age), medium education, medium household income, never-smoker, high-normal glycaemic status (5.4–5.6% HbA1c), and NHANES cycle F (2009–2010).

## Table S2

Generalized Estimating Equations (GEE) model predicting absence of tooth 16 (reference = presence). Coefficients on log scale (*B*, SE, 95% CI) are shown.

| **Fixed effect** | **Estimate** | **SE** | **95%% CI** | ***p*** |
| --- | --- | --- | --- | --- |
| Age: 20–34 | -1.39 | 0.06 | [-1.50, -1.27] | <.001 |
| Age: 60+ | 0.68 | 0.02 | [0.64, 0.72] | <.001 |
| Education attainment: High | -0.51 | 0.03 | [-0.57, -0.45] | <.001 |
| Education attainment: Low | 0.14 | 0.02 | [0.10, 0.17] | <.001 |
| Glycemic status: ≥6.5% | 0.19 | 0.03 | [0.14, 0.24] | <.001 |
| Glycemic status: Low normal <5.4% | -0.08 | 0.03 | [-0.14, -0.02] | .007 |
| Glycemic status: Prediabetes 5.7–6.4% | 0.09 | 0.02 | [0.04, 0.13] | <.001 |
| Household income: High | -0.28 | 0.03 | [-0.34, -0.22] | <.001 |
| Household income: Low | 0.19 | 0.02 | [0.15, 0.22] | <.001 |
| NHANES cycle: G | 0.04 | 0.03 | [-0.02, 0.09] | .194 |
| NHANES cycle: H | 0.09 | 0.03 | [0.03, 0.14] | .002 |
| NHANES cycle: I | -0.06 | 0.03 | [-0.12, -0.01] | .021 |
| NHANES cycle: J | 0.04 | 0.03 | [-0.02, 0.09] | .184 |
| Sex: Male | -0.01 | 0.02 | [-0.05, 0.02] | .494 |
| Smoking: Ever-smoker | 0.36 | 0.02 | [0.32, 0.40] | <.001 |

**Note**. Intercept terms are suppressed from tooth-level GEE tables for clarity, as they represent the baseline log probability of tooth absence in the reference group and are not clinically interpretable. Reference groups: Female (sex), age 35–59 years (age), medium education, medium household income, never-smoker, high-normal glycaemic status (5.4–5.6% HbA1c), and NHANES cycle F (2009–2010).

## Table S3

Generalized Estimating Equations (GEE) model predicting absence of tooth 15 (reference = presence). Coefficients on log scale (*B*, SE, 95% CI) are shown.

| **Fixed effect** | **Estimate** | **SE** | **95%% CI** | ***p*** |
| --- | --- | --- | --- | --- |
| Age: 20–34 | -1.46 | 0.07 | [-1.59, -1.33] | <.001 |
| Age: 60+ | 0.76 | 0.02 | [0.71, 0.80] | <.001 |
| Education attainment: High | -0.51 | 0.04 | [-0.58, -0.44] | <.001 |
| Education attainment: Low | 0.19 | 0.02 | [0.15, 0.23] | <.001 |
| Glycemic status: ≥6.5% | 0.18 | 0.03 | [0.13, 0.24] | <.001 |
| Glycemic status: Low normal <5.4% | -0.09 | 0.03 | [-0.15, -0.03] | .004 |
| Glycemic status: Prediabetes 5.7–6.4% | 0.09 | 0.03 | [0.04, 0.14] | <.001 |
| Household income: High | -0.26 | 0.03 | [-0.33, -0.19] | <.001 |
| Household income: Low | 0.22 | 0.02 | [0.18, 0.27] | <.001 |
| NHANES cycle: G | 0.05 | 0.03 | [-0.01, 0.11] | .123 |
| NHANES cycle: H | 0.07 | 0.03 | [0.01, 0.13] | .014 |
| NHANES cycle: I | -0.02 | 0.03 | [-0.08, 0.04] | .557 |
| NHANES cycle: J | 0.02 | 0.03 | [-0.04, 0.08] | .516 |
| Sex: Male | -0.11 | 0.02 | [-0.15, -0.07] | <.001 |
| Smoking: Ever-smoker | 0.39 | 0.02 | [0.35, 0.43] | <.001 |

**Note**. Intercept terms are suppressed from tooth-level GEE tables for clarity, as they represent the baseline log probability of tooth absence in the reference group and are not clinically interpretable. Reference groups: Female (sex), age 35–59 years (age), medium education, medium household income, never-smoker, high-normal glycaemic status (5.4–5.6% HbA1c), and NHANES cycle F (2009–2010).

## Table S4

Generalized Estimating Equations (GEE) model predicting absence of tooth 14 (reference = presence). Coefficients on log scale (*B*, SE, 95% CI) are shown.

| **Fixed effect** | **Estimate** | **SE** | **95%% CI** | ***p*** |
| --- | --- | --- | --- | --- |
| Age: 20–34 | -0.85 | 0.05 | [-0.94, -0.76] | <.001 |
| Age: 60+ | 0.67 | 0.02 | [0.63, 0.71] | <.001 |
| Education attainment: High | -0.21 | 0.03 | [-0.27, -0.15] | <.001 |
| Education attainment: Low | 0.15 | 0.02 | [0.11, 0.19] | <.001 |
| Glycemic status: ≥6.5% | 0.18 | 0.03 | [0.13, 0.24] | <.001 |
| Glycemic status: Low normal <5.4% | 0.05 | 0.03 | [-0.01, 0.11] | .084 |
| Glycemic status: Prediabetes 5.7–6.4% | 0.06 | 0.03 | [0.01, 0.11] | .014 |
| Household income: High | -0.05 | 0.03 | [-0.11, 0.01] | .091 |
| Household income: Low | 0.25 | 0.02 | [0.20, 0.29] | <.001 |
| NHANES cycle: G | 0.00 | 0.03 | [-0.05, 0.06] | .868 |
| NHANES cycle: H | 0.05 | 0.03 | [-0.01, 0.11] | .092 |
| NHANES cycle: I | -0.05 | 0.03 | [-0.10, 0.01] | .132 |
| NHANES cycle: J | -0.03 | 0.03 | [-0.09, 0.03] | .297 |
| Sex: Male | -0.12 | 0.02 | [-0.16, -0.08] | <.001 |
| Smoking: Ever-smoker | 0.33 | 0.02 | [0.29, 0.37] | <.001 |

**Note**. Intercept terms are suppressed from tooth-level GEE tables for clarity, as they represent the baseline log probability of tooth absence in the reference group and are not clinically interpretable. Reference groups: Female (sex), age 35–59 years (age), medium education, medium household income, never-smoker, high-normal glycaemic status (5.4–5.6% HbA1c), and NHANES cycle F (2009–2010).

## Table S5

Generalized Estimating Equations (GEE) model predicting absence of tooth 13 (reference = presence). Coefficients on log scale (*B*, SE, 95% CI) are shown.

| **Fixed effect** | **Estimate** | **SE** | **95%% CI** | ***p*** |
| --- | --- | --- | --- | --- |
| Age: 20–34 | -1.62 | 0.10 | [-1.82, -1.42] | <.001 |
| Age: 60+ | 1.11 | 0.03 | [1.05, 1.18] | <.001 |
| Education attainment: High | -0.61 | 0.05 | [-0.71, -0.51] | <.001 |
| Education attainment: Low | 0.26 | 0.03 | [0.21, 0.32] | <.001 |
| Glycemic status: ≥6.5% | 0.20 | 0.04 | [0.12, 0.27] | <.001 |
| Glycemic status: Low normal <5.4% | -0.07 | 0.04 | [-0.15, 0.02] | .131 |
| Glycemic status: Prediabetes 5.7–6.4% | 0.09 | 0.04 | [0.02, 0.16] | 0.007 |
| Household income: High | -0.31 | 0.05 | [-0.41, -0.21] | <.001 |
| Household income: Low | 0.34 | 0.03 | [0.28, 0.40] | <.001 |
| NHANES cycle: G | 0.05 | 0.04 | [-0.03, 0.13] | .184 |
| NHANES cycle: H | 0.12 | 0.04 | [0.04, 0.20] | .004 |
| NHANES cycle: I | 0.03 | 0.04 | [-0.05, 0.11] | .497 |
| NHANES cycle: J | 0.07 | 0.04 | [-0.01, 0.15] | .072 |
| Sex: Male | -0.12 | 0.03 | [-0.17, -0.07] | <.001 |
| Smoking: Ever-smoker | 0.51 | 0.03 | [0.46, 0.56] | <.001 |

**Note**. Intercept terms are suppressed from tooth-level GEE tables for clarity, as they represent the baseline log probability of tooth absence in the reference group and are not clinically interpretable. Reference groups: Female (sex), age 35–59 years (age), medium education, medium household income, never-smoker, high-normal glycaemic status (5.4–5.6% HbA1c), and NHANES cycle F (2009–2010).

## Table S6

Generalized Estimating Equations (GEE) model predicting absence of tooth 12 (reference = presence). Coefficients on log scale (*B*, SE, 95% CI) are shown.

| **Fixed effect** | **Estimate** | **SE** | **95%% CI** | ***p*** |
| --- | --- | --- | --- | --- |
| Age: 20–34 | -1.55 | 0.09 | [-1.72, -1.38] | <.001 |
| Age: 60+ | 0.95 | 0.03 | [0.90, 1.01] | <.001 |
| Education attainment: High | -0.53 | 0.04 | [-0.62, -0.44] | <.001 |
| Education attainment: Low | 0.26 | 0.02 | [0.22, 0.31] | <.001 |
| Glycemic status: ≥6.5% | 0.18 | 0.04 | [0.11, 0.24] | <.001 |
| Glycemic status: Low normal <5.4% | -0.04 | 0.04 | [-0.12, 0.03] | .248 |
| Glycemic status: Prediabetes 5.7–6.4% | 0.09 | 0.03 | [0.03, 0.15] | .004 |
| Household income: High | -0.30 | 0.04 | [-0.38, -0.22] | <.001 |
| Household income: Low | 0.29 | 0.03 | [0.24, 0.35] | <.001 |
| NHANES cycle: G | 0.07 | 0.04 | [-0.00, 0.14] | .061 |
| NHANES cycle: H | 0.09 | 0.04 | [0.02, 0.16] | .015 |
| NHANES cycle: I | -0.02 | 0.04 | [-0.09, 0.05] | .610 |
| NHANES cycle: J | 0.03 | 0.04 | [-0.04, 0.10] | .373 |
| Sex: Male | -0.01 | 0.02 | [-0.05, 0.04] | .789 |
| Smoking: Ever-smoker | 0.46 | 0.02 | [0.41, 0.51] | <.001 |

**Note**. Intercept terms are suppressed from tooth-level GEE tables for clarity, as they represent the baseline log probability of tooth absence in the reference group and are not clinically interpretable. Reference groups: Female (sex), age 35–59 years (age), medium education, medium household income, never-smoker, high-normal glycaemic status (5.4–5.6% HbA1c), and NHANES cycle F (2009–2010).

## Table S7

Generalized Estimating Equations (GEE) model predicting absence of tooth 11 (reference = presence). Coefficients on log scale (*B*, SE, 95% CI) are shown.

| **Fixed effect** | **Estimate** | **SE** | **95%% CI** | **p** |
| --- | --- | --- | --- | --- |
| Age: 20–34 | -1.83 | 0.10 | [-2.03, -1.64] | <.001 |
| Age: 60+ | 0.97 | 0.03 | [0.92, 1.03] | <.001 |
| Education attainment: High | -0.54 | 0.04 | [-0.62, -0.45] | <.001 |
| Education attainment: Low | 0.29 | 0.02 | [0.25, 0.34] | <.001 |
| Glycemic status: ≥6.5% | 0.19 | 0.04 | [0.12, 0.26] | <.001 |
| Glycemic status: Low normal <5.4% | -0.07 | 0.04 | [-0.15, 0.00] | .066 |
| Glycemic status: Prediabetes 5.7–6.4% | 0.08 | 0.03 | [0.02, 0.14] | .008 |
| Household income: High | -0.28 | 0.04 | [-0.36, -0.20] | <.001 |
| Household income: Low | 0.33 | 0.03 | [0.27, 0.38] | <.001 |
| NHANES cycle: G | 0.10 | 0.04 | [0.02, 0.17] | .010 |
| NHANES cycle: H | 0.11 | 0.04 | [0.04, 0.18] | .003 |
| NHANES cycle: I | 0.02 | 0.04 | [-0.05, 0.09] | .590 |
| NHANES cycle: J | 0.00 | 0.04 | [-0.07, 0.08] | .888 |
| Sex: Male | 0.01 | 0.02 | [-0.03, 0.06] | .537 |
| Smoking: Ever-smoker | 0.44 | 0.02 | [0.39, 0.49] | <.001 |

**Note**. Intercept terms are suppressed from tooth-level GEE tables for clarity, as they represent the baseline log probability of tooth absence in the reference group and are not clinically interpretable. Reference groups: Female (sex), age 35–59 years (age), medium education, medium household income, never-smoker, high-normal glycaemic status (5.4–5.6% HbA1c), and NHANES cycle F (2009–2010).

## Table S8

Generalized Estimating Equations (GEE) model predicting absence of tooth 21 (reference = presence). Coefficients on log scale (*B*, SE, 95% CI) are shown.

| **Fixed effect** | **Estimate** | **SE** | **95%% CI** | ***p*** |
| --- | --- | --- | --- | --- |
| Age: 20–34 | -1.78 | 0.10 | [-1.97, -1.59] | <.001 |
| Age: 60+ | 0.98 | 0.03 | [0.92, 1.04] | <.001 |
| Education attainment: High | -0.55 | 0.04 | [-0.64, -0.46] | <.001 |
| Education attainment: Low | 0.27 | 0.02 | [0.23, 0.32] | <.001 |
| Glycemic status: ≥6.5% | 0.20 | 0.04 | [0.13, 0.27] | <.001 |
| Glycemic status: Low normal <5.4% | -0.05 | 0.04 | [-0.12, 0.03] | .224 |
| Glycemic status: Prediabetes 5.7–6.4% | 0.08 | 0.03 | [0.02, 0.14] | .008 |
| Household income: High | -0.27 | 0.04 | [-0.35, -0.18] | <.001 |
| Household income: Low | 0.32 | 0.03 | [0.26, 0.38] | <.001 |
| NHANES cycle: G | 0.03 | 0.04 | [-0.04, 0.10] | .458 |
| NHANES cycle: H | 0.07 | 0.04 | [0.00, 0.15] | .046 |
| NHANES cycle: I | -0.02 | 0.04 | [-0.10, 0.05] | .559 |
| NHANES cycle: J | -0.02 | 0.04 | [-0.09, 0.06] | .668 |
| Sex: Male | 0.01 | 0.02 | [-0.03, 0.06] | .598 |
| Smoking: Ever-smoker | 0.45 | 0.02 | [0.40, 0.50] | <.001 |

**Note**. Intercept terms are suppressed from tooth-level GEE tables for clarity, as they represent the baseline log probability of tooth absence in the reference group and are not clinically interpretable. Reference groups: Female (sex), age 35–59 years (age), medium education, medium household income, never-smoker, high-normal glycaemic status (5.4–5.6% HbA1c), and NHANES cycle F (2009–2010).

## Table S9

Generalized Estimating Equations (GEE) model predicting absence of tooth 22 (reference = presence). Coefficients on log scale (*B*, SE, 95% CI) are shown.

| **Fixed effect** | **Estimate** | **SE** | **95%% CI** | ***p*** |
| --- | --- | --- | --- | --- |
| Age: 20–34 | -1.54 | 0.09 | [-1.71, -1.37] | <.001 |
| Age: 60+ | 0.96 | 0.03 | [0.90, 1.01] | <.001 |
| Education attainment: High | -0.56 | 0.04 | [-0.65, -0.47] | <.001 |
| Education attainment: Low | 0.27 | 0.02 | [0.23, 0.32] | <.001 |
| Glycemic status: ≥6.5% | 0.15 | 0.04 | [0.09, 0.22] | <.001 |
| Glycemic status: Low normal <5.4% | -0.08 | 0.04 | [-0.15, -0.00] | .050 |
| Glycemic status: Prediabetes 5.7–6.4% | 0.07 | 0.03 | [0.01, 0.14] | .015 |
| Household income: High | -0.31 | 0.04 | [-0.40, -0.22] | <.001 |
| Household income: Low | 0.30 | 0.03 | [0.24, 0.35] | <.001 |
| NHANES cycle: G | 0.05 | 0.04 | [-0.03, 0.12] | .209 |
| NHANES cycle: H | 0.06 | 0.04 | [-0.01, 0.13] | .094 |
| NHANES cycle: I | -0.04 | 0.04 | [-0.11, 0.03] | .296 |
| NHANES cycle: J | 0.03 | 0.04 | [-0.04, 0.10] | .464 |
| Sex: Male | -0.02 | 0.02 | [-0.07, 0.03] | .416 |
| Smoking: Ever-smoker | 0.45 | 0.02 | [0.40, 0.50] | <.001 |

**Note**. Intercept terms are suppressed from tooth-level GEE tables for clarity, as they represent the baseline log probability of tooth absence in the reference group and are not clinically interpretable. Reference groups: Female (sex), age 35–59 years (age), medium education, medium household income, never-smoker, high-normal glycaemic status (5.4–5.6% HbA1c), and NHANES cycle F (2009–2010).

## Table S10

Generalized Estimating Equations (GEE) model predicting absence of tooth 23 (reference = presence). Coefficients on log scale (*B*, SE, 95% CI) are shown.

| **Fixed effect** | **Estimate** | **SE** | **95%% CI** | ***p*** |
| --- | --- | --- | --- | --- |
| Age: 20–34 | -1.65 | 0.10 | [-1.85, -1.45] | <.001 |
| Age: 60+ | 1.09 | 0.03 | [1.02, 1.15] | <.001 |
| Education attainment: High | -0.64 | 0.05 | [-0.74, -0.54] | <.001 |
| Education attainment: Low | 0.26 | 0.03 | [0.21, 0.31] | <.001 |
| Glycemic status: ≥6.5% | 0.23 | 0.04 | [0.16, 0.31] | <.001 |
| Glycemic status: Low normal <5.4% | -0.02 | 0.04 | [-0.11, 0.06] | .626 |
| Glycemic status: Prediabetes 5.7–6.4% | 0.11 | 0.04 | [0.04, 0.18] | .001 |
| Household income: High | -0.36 | 0.05 | [-0.45, -0.26] | <.001 |
| Household income: Low | 0.32 | 0.03 | [0.26, 0.38] | <.001 |
| NHANES cycle: G | 0.03 | 0.04 | [-0.05, 0.11] | .402 |
| NHANES cycle: H | 0.09 | 0.04 | [0.01, 0.17] | .028 |
| NHANES cycle: I | 0.01 | 0.04 | [-0.07, 0.09] | .827 |
| NHANES cycle: J | 0.06 | 0.04 | [-0.02, 0.14] | .130 |
| Sex: Male | -0.09 | 0.03 | [-0.14, -0.04] | <.001 |
| Smoking: Ever-smoker | 0.53 | 0.03 | [0.48, 0.59] | <.001 |

**Note**. Intercept terms are suppressed from tooth-level GEE tables for clarity, as they represent the baseline log probability of tooth absence in the reference group and are not clinically interpretable. Reference groups: Female (sex), age 35–59 years (age), medium education, medium household income, never-smoker, high-normal glycaemic status (5.4–5.6% HbA1c), and NHANES cycle F (2009–2010).

## Table S11

Generalized Estimating Equations (GEE) model predicting absence of tooth 24 (reference = presence). Coefficients on log scale (*B*, SE, 95% CI) are shown.

| **Fixed effect** | **Estimate** | **SE** | **95%% CI** | ***p*** |
| --- | --- | --- | --- | --- |
| Age: 20–34 | -0.86 | 0.05 | [-0.95, -0.76] | <.001 |
| Age: 60+ | 0.65 | 0.02 | [0.61, 0.69] | <.001 |
| Education attainment: High | -0.21 | 0.03 | [-0.27, -0.15] | <.001 |
| Education attainment: Low | 0.14 | 0.02 | [0.10, 0.18] | <.001 |
| Glycemic status: ≥6.5% | 0.19 | 0.03 | [0.13, 0.24] | <.001 |
| Glycemic status: Low normal <5.4% | 0.03 | 0.03 | [-0.03, 0.08] | .384 |
| Glycemic status: Prediabetes 5.7–6.4% | 0.05 | 0.03 | [-0.00, 0.10] | .073 |
| Household income: High | -0.09 | 0.03 | [-0.15, -0.03] | .003 |
| Household income: Low | 0.22 | 0.02 | [0.18, 0.27] | <.001 |
| NHANES cycle: G | -0.01 | 0.03 | [-0.07, 0.05] | .772 |
| NHANES cycle: H | 0.06 | 0.03 | [-0.00, 0.12] | .051 |
| NHANES cycle: I | -0.04 | 0.03 | [-0.09, 0.02] | .250 |
| NHANES cycle: J | -0.04 | 0.03 | [-0.10, 0.02] | .214 |
| Sex: Male | -0.12 | 0.02 | [-0.16, -0.08] | <.001 |
| Smoking: Ever-smoker | 0.31 | 0.02 | [0.27, 0.35] | <.001 |

**Note**. Intercept terms are suppressed from tooth-level GEE tables for clarity, as they represent the baseline log probability of tooth absence in the reference group and are not clinically interpretable. Reference groups: Female (sex), age 35–59 years (age), medium education, medium household income, never-smoker, high-normal glycaemic status (5.4–5.6% HbA1c), and NHANES cycle F (2009–2010).

## Table S12

Generalized Estimating Equations (GEE) model predicting absence of tooth 25 (reference = presence). Coefficients on log scale (*B*, SE, 95% CI) are shown.

| **Fixed effect** | **Estimate** | **SE** | **95%% CI** | ***p*** |
| --- | --- | --- | --- | --- |
| Age: 20–34 | -1.42 | 0.07 | [-1.55, -1.29] | <.001 |
| Age: 60+ | 0.78 | 0.02 | [0.73, 0.82] | <.001 |
| Education attainment: High | -0.52 | 0.04 | [-0.59, -0.45] | <.001 |
| Education attainment: Low | 0.15 | 0.02 | [0.11, 0.19] | <.001 |
| Glycemic status: ≥6.5% | 0.18 | 0.03 | [0.13, 0.24] | <.001 |
| Glycemic status: Low normal <5.4% | -0.06 | 0.03 | [-0.13, -0.00] | .045 |
| Glycemic status: Prediabetes 5.7–6.4% | 0.09 | 0.03 | [0.04, 0.14] | <.001 |
| Household income: High | -0.30 | 0.04 | [-0.37, -0.23] | <.001 |
| Household income: Low | 0.24 | 0.02 | [0.20, 0.28] | <.001 |
| NHANES cycle: G | 0.05 | 0.03 | [-0.01, 0.11] | .079 |
| NHANES cycle: H | 0.10 | 0.03 | [0.05, 0.16] | <.001 |
| NHANES cycle: I | -0.03 | 0.03 | [-0.09, 0.03] | .391 |
| NHANES cycle: J | 0.03 | 0.03 | [-0.03, 0.09] | .316 |
| Sex: Male | -0.11 | 0.02 | [-0.14, -0.07] | <.001 |
| Smoking: Ever-smoker | 0.42 | 0.02 | [0.38, 0.46] | <.001 |

**Note**. Intercept terms are suppressed from tooth-level GEE tables for clarity, as they represent the baseline log probability of tooth absence in the reference group and are not clinically interpretable. Reference groups: Female (sex), age 35–59 years (age), medium education, medium household income, never-smoker, high-normal glycaemic status (5.4–5.6% HbA1c), and NHANES cycle F (2009–2010).

## Table S13

Generalized Estimating Equations (GEE) model predicting absence of tooth 26 (reference = presence). Coefficients on log scale (*B*, SE, 95% CI) are shown.

| **Fixed effect** | **Estimate** | **SE** | **95%% CI** | ***p*** |
| --- | --- | --- | --- | --- |
| Age: 20–34 | -1.38 | 0.06 | [-1.49, -1.27] | <.001 |
| Age: 60+ | 0.65 | 0.02 | [0.61, 0.69] | <.001 |
| Education attainment: High | -0.52 | 0.03 | [-0.58, -0.45] | <.001 |
| Education attainment: Low | 0.15 | 0.02 | [0.11, 0.18] | <.001 |
| Glycemic status: ≥6.5% | 0.18 | 0.03 | [0.14, 0.23] | <.001 |
| Glycemic status: Low normal <5.4% | -0.06 | 0.03 | [-0.12, -0.01] | .026 |
| Glycemic status: Prediabetes 5.7–6.4% | 0.07 | 0.02 | [0.03, 0.12] | .002 |
| Household income: High | -0.32 | 0.03 | [-0.38, -0.26] | <.001 |
| Household income: Low | 0.17 | 0.02 | [0.14, 0.21] | <.001 |
| NHANES cycle: G | 0.03 | 0.03 | [-0.02, 0.09] | .216 |
| NHANES cycle: H | 0.06 | 0.03 | [0.01, 0.12] | .016 |
| NHANES cycle: I | -0.03 | 0.03 | [-0.09, 0.02] | .253 |
| NHANES cycle: J | 0.01 | 0.03 | [-0.04, 0.06] | .673 |
| Sex: Male | -0.05 | 0.02 | [-0.08, -0.01] | .007 |
| Smoking: Ever-smoker | 0.38 | 0.02 | [0.35, 0.42] | <.001 |

**Note**. Intercept terms are suppressed from tooth-level GEE tables for clarity, as they represent the baseline log probability of tooth absence in the reference group and are not clinically interpretable. Reference groups: Female (sex), age 35–59 years (age), medium education, medium household income, never-smoker, high-normal glycaemic status (5.4–5.6% HbA1c), and NHANES cycle F (2009–2010).

## Table S14

Generalized Estimating Equations (GEE) model predicting absence of tooth 27 (reference = presence). Coefficients on log scale (*B*, SE, 95% CI) are shown.

| **Fixed effect** | **Estimate** | **SE** | **95%% CI** | ***p*** |
| --- | --- | --- | --- | --- |
| Age: 20–34 | -1.34 | 0.06 | [-1.46, -1.23] | <.001 |
| Age: 60+ | 0.70 | 0.02 | [0.66, 0.74] | <.001 |
| Education attainment: High | -0.53 | 0.03 | [-0.59, -0.46] | <.001 |
| Education attainment: Low | 0.11 | 0.02 | [0.07, 0.14] | <.001 |
| Glycemic status: ≥6.5% | 0.20 | 0.03 | [0.15, 0.25] | <.001 |
| Glycemic status: Low normal <5.4% | -0.03 | 0.03 | [-0.09, 0.02] | .264 |
| Glycemic status: Prediabetes 5.7–6.4% | 0.12 | 0.02 | [0.07, 0.17] | <.001 |
| Household income: High | -0.23 | 0.03 | [-0.29, -0.17] | <.001 |
| Household income: Low | 0.20 | 0.02 | [0.16, 0.24] | <.001 |
| NHANES cycle: G | 0.04 | 0.03 | [-0.01, 0.10] | .148 |
| NHANES cycle: H | 0.11 | 0.03 | [0.05, 0.16] | <.001 |
| NHANES cycle: I | 0.01 | 0.03 | [-0.04, 0.07] | .605 |
| NHANES cycle: J | 0.08 | 0.03 | [0.03, 0.14] | .002 |
| Sex: Male | -0.10 | 0.02 | [-0.13, -0.06] | <.001 |
| Smoking: Ever-smoker | 0.44 | 0.02 | [0.40, 0.47] | <.001 |

**Note**. Intercept terms are suppressed from tooth-level GEE tables for clarity, as they represent the baseline log probability of tooth absence in the reference group and are not clinically interpretable. Reference groups: Female (sex), age 35–59 years (age), medium education, medium household income, never-smoker, high-normal glycaemic status (5.4–5.6% HbA1c), and NHANES cycle F (2009–2010).

## Table S15

Generalized Estimating Equations (GEE) model predicting absence of tooth 37 (reference = presence). Coefficients on log scale (*B*, SE, 95% CI) are shown.

| **Fixed effect** | **Estimate** | **SE** | **95%% CI** | ***p*** |
| --- | --- | --- | --- | --- |
| Age: 20–34 | -1.15 | 0.05 | [-1.24, -1.05] | <.001 |
| Age: 60+ | 0.61 | 0.02 | [0.58, 0.65] | <.001 |
| Education attainment: High | -0.52 | 0.03 | [-0.58, -0.46] | <.001 |
| Education attainment: Low | 0.16 | 0.02 | [0.13, 0.19] | <.001 |
| Glycemic status: ≥6.5% | 0.16 | 0.02 | [0.11, 0.21] | <.001 |
| Glycemic status: Low normal <5.4% | -0.13 | 0.03 | [-0.18, -0.07] | <.001 |
| Glycemic status: Prediabetes 5.7–6.4% | 0.06 | 0.02 | [0.02, 0.10] | .003 |
| Household income: High | -0.26 | 0.03 | [-0.31, -0.20] | <.001 |
| Household income: Low | 0.20 | 0.02 | [0.16, 0.23] | <.001 |
| NHANES cycle: G | 0.00 | 0.02 | [-0.05, 0.05] | .989 |
| NHANES cycle: H | 0.09 | 0.02 | [0.04, 0.14] | <.001 |
| NHANES cycle: I | 0.00 | 0.02 | [-0.04, 0.05] | .863 |
| NHANES cycle: J | 0.00 | 0.02 | [-0.05, 0.05] | .938 |
| Sex: Male | -0.04 | 0.02 | [-0.08, -0.01] | .006 |
| Smoking: Ever-smoker | 0.30 | 0.02 | [0.27, 0.34] | <.001 |

**Note**. Intercept terms are suppressed from tooth-level GEE tables for clarity, as they represent the baseline log probability of tooth absence in the reference group and are not clinically interpretable. Reference groups: Female (sex), age 35–59 years (age), medium education, medium household income, never-smoker, high-normal glycaemic status (5.4–5.6% HbA1c), and NHANES cycle F (2009–2010).

## Table S16

Generalized Estimating Equations (GEE) model predicting absence of tooth 36 (reference = presence). Coefficients on log scale (*B*, SE, 95% CI) are shown.

| **Fixed effect** | **Estimate** | **SE** | **95%% CI** | ***p*** |
| --- | --- | --- | --- | --- |
| Age: 20–34 | -1.15 | 0.04 | [-1.23, -1.06] | <.001 |
| Age: 60+ | 0.50 | 0.02 | [0.47, 0.53] | <.001 |
| Education attainment: High | -0.48 | 0.03 | [-0.53, -0.42] | <.001 |
| Education attainment: Low | 0.13 | 0.01 | [0.10, 0.16] | <.001 |
| Glycemic status: ≥6.5% | 0.14 | 0.02 | [0.10, 0.18] | <.001 |
| Glycemic status: Low normal <5.4% | -0.11 | 0.02 | [-0.16, -0.06] | <.001 |
| Glycemic status: Prediabetes 5.7–6.4% | 0.08 | 0.02 | [0.04, 0.12] | <.001 |
| Household income: High | -0.24 | 0.02 | [-0.29, -0.19] | <.001 |
| Household income: Low | 0.15 | 0.02 | [0.12, 0.19] | <.001 |
| NHANES cycle: G | -0.00 | 0.02 | [-0.05, 0.04] | .921 |
| NHANES cycle: H | 0.02 | 0.02 | [-0.03, 0.06] | .468 |
| NHANES cycle: I | -0.02 | 0.02 | [-0.06, 0.02] | .386 |
| NHANES cycle: J | -0.02 | 0.02 | [-0.06, 0.03] | .495 |
| Sex: Male | -0.06 | 0.01 | [-0.09, -0.03] | <.001 |
| Smoking: Ever-smoker | 0.22 | 0.01 | [0.19, 0.25] | <.001 |

**Note**. Intercept terms are suppressed from tooth-level GEE tables for clarity, as they represent the baseline log probability of tooth absence in the reference group and are not clinically interpretable. Reference groups: Female (sex), age 35–59 years (age), medium education, medium household income, never-smoker, high-normal glycaemic status (5.4–5.6% HbA1c), and NHANES cycle F (2009–2010).

## Table S17

Generalized Estimating Equations (GEE) model predicting absence of tooth 35 (reference = presence). Coefficients on log scale (*B*, SE, 95% CI) are shown.

| **Fixed effect** | **Estimate** | **SE** | **95%% CI** | ***p*** |
| --- | --- | --- | --- | --- |
| Age: 20–34 | -1.30 | 0.07 | [-1.45, -1.16] | <.001 |
| Age: 60+ | 0.92 | 0.03 | [0.87, 0.97] | <.001 |
| Education attainment: High | -0.47 | 0.04 | [-0.55, -0.38] | <.001 |
| Education attainment: Low | 0.17 | 0.02 | [0.13, 0.22] | <.001 |
| Glycemic status: ≥6.5% | 0.19 | 0.03 | [0.13, 0.26] | <.001 |
| Glycemic status: Low normal <5.4% | -0.09 | 0.04 | [-0.16, -0.01] | .020 |
| Glycemic status: Prediabetes 5.7–6.4% | 0.08 | 0.03 | [0.02, 0.14] | .007 |
| Household income: High | -0.28 | 0.04 | [-0.36, -0.20] | <.001 |
| Household income: Low | 0.27 | 0.03 | [0.22, 0.32] | <.001 |
| NHANES cycle: G | 0.02 | 0.04 | [-0.05, 0.09] | .609 |
| NHANES cycle: H | 0.07 | 0.04 | [0.00, 0.14] | .042 |
| NHANES cycle: I | -0.03 | 0.04 | [-0.10, 0.04] | .354 |
| NHANES cycle: J | 0.02 | 0.04 | [-0.05, 0.09] | .627 |
| Sex: Male | -0.08 | 0.02 | [-0.12, -0.03] | <.001 |
| Smoking: Ever-smoker | 0.40 | 0.02 | [0.35, 0.45] | <.001 |

**Note**. Intercept terms are suppressed from tooth-level GEE tables for clarity, as they represent the baseline log probability of tooth absence in the reference group and are not clinically interpretable. Reference groups: Female (sex), age 35–59 years (age), medium education, medium household income, never-smoker, high-normal glycaemic status (5.4–5.6% HbA1c), and NHANES cycle F (2009–2010).

## Table S18

Generalized Estimating Equations (GEE) model predicting absence of tooth 34 (reference = presence). Coefficients on log scale (*B*, SE, 95% CI) are shown.

| **Fixed effect** | **Estimate** | **SE** | **95%% CI** | ***p*** |
| --- | --- | --- | --- | --- |
| Age: 20–34 | -0.70 | 0.06 | [-0.82, -0.59] | <.001 |
| Age: 60+ | 0.83 | 0.03 | [0.77, 0.89] | <.001 |
| Education attainment: High | -0.16 | 0.04 | [-0.24, -0.08] | <.001 |
| Education attainment: Low | 0.19 | 0.03 | [0.14, 0.25] | <.001 |
| Glycemic status: ≥6.5% | 0.15 | 0.04 | [0.07, 0.23] | <.001 |
| Glycemic status: Low normal <5.4% | 0.06 | 0.04 | [-0.02, 0.14] | .115 |
| Glycemic status: Prediabetes 5.7–6.4% | 0.03 | 0.04 | [-0.04, 0.10] | .373 |
| Household income: High | -0.01 | 0.04 | [-0.09, 0.07] | .838 |
| Household income: Low | 0.26 | 0.03 | [0.20, 0.32] | <.001 |
| NHANES cycle: G | 0.03 | 0.04 | [-0.06, 0.11] | .520 |
| NHANES cycle: H | 0.05 | 0.04 | [-0.04, 0.13] | .255 |
| NHANES cycle: I | -0.09 | 0.04 | [-0.17, -0.00] | .047 |
| NHANES cycle: J | -0.03 | 0.04 | [-0.11, 0.06] | .511 |
| Sex: Male | -0.09 | 0.03 | [-0.14, -0.04] | .001 |
| Smoking: Ever-smoker | 0.35 | 0.03 | [0.30, 0.40] | <.001 |

**Note**. Intercept terms are suppressed from tooth-level GEE tables for clarity, as they represent the baseline log probability of tooth absence in the reference group and are not clinically interpretable. Reference groups: Female (sex), age 35–59 years (age), medium education, medium household income, never-smoker, high-normal glycaemic status (5.4–5.6% HbA1c), and NHANES cycle F (2009–2010).

## Table S19

Generalized Estimating Equations (GEE) model predicting absence of tooth 33 (reference = presence). Coefficients on log scale (*B*, SE, 95% CI) are shown.

| **Fixed effect** | **Estimate** | **SE** | **95%% CI** | ***p*** |
| --- | --- | --- | --- | --- |
| Age: 20–34 | -2.08 | 0.19 | [-2.45, -1.72] | <.001 |
| Age: 60+ | 1.34 | 0.05 | [1.25, 1.44] | <.001 |
| Education attainment: High | -0.84 | 0.08 | [-1.00, -0.69] | <.001 |
| Education attainment: Low | 0.34 | 0.04 | [0.26, 0.41] | <.001 |
| Glycemic status: ≥6.5% | 0.19 | 0.06 | [0.08, 0.29] | <.001 |
| Glycemic status: Low normal <5.4% | -0.09 | 0.06 | [-0.22, 0.03] | .140 |
| Glycemic status: Prediabetes 5.7–6.4% | 0.08 | 0.05 | [-0.01, 0.18] | .088 |
| Household income: High | -0.33 | 0.07 | [-0.47, -0.19] | <.001 |
| Household income: Low | 0.41 | 0.04 | [0.32, 0.50] | <.001 |
| NHANES cycle: G | 0.08 | 0.06 | [-0.04, 0.19] | .181 |
| NHANES cycle: H | 0.13 | 0.06 | [0.01, 0.24] | .026 |
| NHANES cycle: I | -0.04 | 0.06 | [-0.15, 0.08] | .535 |
| NHANES cycle: J | 0.08 | 0.06 | [-0.03, 0.19] | .155 |
| Sex: Male | -0.02 | 0.04 | [-0.10, 0.05] | .535 |
| Smoking: Ever-smoker | 0.57 | 0.04 | [0.50, 0.65] | <.001 |

**Note**. Intercept terms are suppressed from tooth-level GEE tables for clarity, as they represent the baseline log probability of tooth absence in the reference group and are not clinically interpretable. Reference groups: Female (sex), age 35–59 years (age), medium education, medium household income, never-smoker, high-normal glycaemic status (5.4–5.6% HbA1c), and NHANES cycle F (2009–2010).

## Table S20

Generalized Estimating Equations (GEE) model predicting absence of tooth 32 (reference = presence). Coefficients on log scale (*B*, SE, 95% CI) are shown.

| **Fixed effect** | **Estimate** | **SE** | **95%% CI** | ***p*** |
| --- | --- | --- | --- | --- |
| Age: 20–34 | -2.05 | 0.16 | [-2.37, -1.74] | <.001 |
| Age: 60+ | 1.27 | 0.04 | [1.19, 1.36] | <.001 |
| Education attainment: High | -0.62 | 0.06 | [-0.75, -0.49] | <.001 |
| Education attainment: Low | 0.30 | 0.03 | [0.23, 0.37] | <.001 |
| Glycemic status: ≥6.5% | 0.25 | 0.05 | [0.15, 0.34] | <.001 |
| Glycemic status: Low normal <5.4% | -0.06 | 0.06 | [-0.17, 0.05] | .263 |
| Glycemic status: Prediabetes 5.7–6.4% | 0.10 | 0.04 | [0.02, 0.19] | .019 |
| Household income: High | -0.28 | 0.06 | [-0.40, -0.16] | <.001 |
| Household income: Low | 0.37 | 0.04 | [0.29, 0.45] | <.001 |
| NHANES cycle: G | 0.14 | 0.05 | [0.04, 0.23] | .007 |
| NHANES cycle: H | 0.13 | 0.05 | [0.03, 0.23] | .012 |
| NHANES cycle: I | -0.03 | 0.05 | [-0.13, 0.08] | .605 |
| NHANES cycle: J | 0.07 | 0.05 | [-0.03, 0.17] | .152 |
| Sex: Male | 0.07 | 0.03 | [0.01, 0.14] | .032 |
| Smoking: Ever-smoker | 0.56 | 0.04 | [0.49, 0.63] | <.001 |

**Note**. Intercept terms are suppressed from tooth-level GEE tables for clarity, as they represent the baseline log probability of tooth absence in the reference group and are not clinically interpretable. Reference groups: Female (sex), age 35–59 years (age), medium education, medium household income, never-smoker, high-normal glycaemic status (5.4–5.6% HbA1c), and NHANES cycle F (2009–2010).

## Table S21

Generalized Estimating Equations (GEE) model predicting absence of tooth 31 (reference = presence). Coefficients on log scale (*B*, SE, 95% CI) are shown.

| **Fixed effect** | **Estimate** | **SE** | **95%% CI** | ***p*** |
| --- | --- | --- | --- | --- |
| Age: 20–34 | -1.88 | 0.14 | [-2.15, -1.60] | <.001 |
| Age: 60+ | 1.24 | 0.04 | [1.16, 1.31] | <.001 |
| Education attainment: High | -0.62 | 0.06 | [-0.74, -0.50] | <.001 |
| Education attainment: Low | 0.31 | 0.03 | [0.25, 0.37] | <.001 |
| Glycemic status: ≥6.5% | 0.24 | 0.05 | [0.15, 0.33] | <.001 |
| Glycemic status: Low normal <5.4% | -0.07 | 0.05 | [-0.17, 0.04] | .206 |
| Glycemic status: Prediabetes 5.7–6.4% | 0.10 | 0.04 | [0.02, 0.18] | .012 |
| Household income: High | -0.26 | 0.06 | [-0.37, -0.15] | <.001 |
| Household income: Low | 0.34 | 0.04 | [0.27, 0.41] | <.001 |
| NHANES cycle: G | 0.11 | 0.05 | [0.02, 0.20] | .022 |
| NHANES cycle: H | 0.11 | 0.05 | [0.01, 0.20] | .027 |
| NHANES cycle: I | -0.00 | 0.05 | [-0.10, 0.09] | .948 |
| NHANES cycle: J | 0.06 | 0.05 | [-0.03, 0.16] | .171 |
| Sex: Male | 0.09 | 0.03 | [0.03, 0.15] | .002 |
| Smoking: Ever-smoker | 0.55 | 0.03 | [0.48, 0.61] | <.001 |

**Note**. Intercept terms are suppressed from tooth-level GEE tables for clarity, as they represent the baseline log probability of tooth absence in the reference group and are not clinically interpretable. Reference groups: Female (sex), age 35–59 years (age), medium education, medium household income, never-smoker, high-normal glycaemic status (5.4–5.6% HbA1c), and NHANES cycle F (2009–2010).

## Table S22

Generalized Estimating Equations (GEE) model predicting absence of tooth 41 (reference = presence). Coefficients on log scale (*B*, SE, 95% CI) are shown.

| **Fixed effect** | **Estimate** | **SE** | **95%% CI** | ***p*** |
| --- | --- | --- | --- | --- |
| Age: 20–34 | -2.00 | 0.15 | [-2.28, -1.71] | <.001 |
| Age: 60+ | 1.23 | 0.04 | [1.15, 1.31] | <.001 |
| Education attainment: High | -0.60 | 0.06 | [-0.71, -0.48] | <.001 |
| Education attainment: Low | 0.33 | 0.03 | [0.27, 0.39] | <.001 |
| Glycemic status: ≥6.5% | 0.21 | 0.04 | [0.12, 0.30] | <.001 |
| Glycemic status: Low normal <5.4% | -0.09 | 0.05 | [-0.19, 0.01] | .071 |
| Glycemic status: Prediabetes 5.7–6.4% | 0.08 | 0.04 | [0.00, 0.16] | .042 |
| Household income: High | -0.22 | 0.06 | [-0.33, -0.11] | <.001 |
| Household income: Low | 0.36 | 0.04 | [0.29, 0.44] | <.001 |
| NHANES cycle: G | 0.10 | 0.05 | [0.01, 0.20] | .031 |
| NHANES cycle: H | 0.11 | 0.05 | [0.02, 0.20] | .021 |
| NHANES cycle: I | 0.00 | 0.05 | [-0.09, 0.10] | .975 |
| NHANES cycle: J | 0.04 | 0.05 | [-0.06, 0.13] | .446 |
| Sex: Male | 0.10 | 0.03 | [0.03, 0.15] | .002 |
| Smoking: Ever-smoker | 0.50 | 0.03 | [0.44, 0.57] | <.001 |

**Note**. Intercept terms are suppressed from tooth-level GEE tables for clarity, as they represent the baseline log probability of tooth absence in the reference group and are not clinically interpretable. Reference groups: Female (sex), age 35–59 years (age), medium education, medium household income, never-smoker, high-normal glycaemic status (5.4–5.6% HbA1c), and NHANES cycle F (2009–2010).

## Table S23

Generalized Estimating Equations (GEE) model predicting absence of tooth 42 (reference = presence). Coefficients on log scale (*B*, SE, 95% CI) are shown.

| **Fixed effect** | **Estimate** | **SE** | **95%% CI** | ***p*** |
| --- | --- | --- | --- | --- |
| Age: 20–34 | -1.81 | 0.14 | [-2.09, -1.53] | <.001 |
| Age: 60+ | 1.26 | 0.04 | [1.17, 1.34] | <.001 |
| Education attainment: High | -0.68 | 0.07 | [-0.81, -0.55] | <.001 |
| Education attainment: Low | 0.32 | 0.03 | [0.25, 0.38] | <.001 |
| Glycemic status: ≥6.5% | 0.26 | 0.05 | [0.16, 0.35] | <.001 |
| Glycemic status: Low normal <5.4% | -0.06 | 0.06 | [-0.17, 0.05] | .264 |
| Glycemic status: Prediabetes 5.7–6.4% | 0.10 | 0.04 | [0.02, 0.19] | .016 |
| Household income: High | -0.24 | 0.06 | [-0.36, -0.12] | <.001 |
| Household income: Low | 0.38 | 0.04 | [0.30, 0.45] | <.001 |
| NHANES cycle: G | 0.11 | 0.05 | [0.01, 0.21] | .026 |
| NHANES cycle: H | 0.13 | 0.05 | [0.03, 0.23] | .009 |
| NHANES cycle: I | -0.05 | 0.05 | [-0.15, 0.06] | .373 |
| NHANES cycle: J | 0.05 | 0.05 | [-0.05, 0.15] | .345 |
| Sex: Male | 0.06 | 0.03 | [-0.00, 0.13] | .050 |
| Smoking: Ever-smoker | 0.55 | 0.04 | [0.48, 0.61] | <.001 |

**Note**. Intercept terms are suppressed from tooth-level GEE tables for clarity, as they represent the baseline log probability of tooth absence in the reference group and are not clinically interpretable. Reference groups: Female (sex), age 35–59 years (age), medium education, medium household income, never-smoker, high-normal glycaemic status (5.4–5.6% HbA1c), and NHANES cycle F (2009–2010).

## Table S24

Generalized Estimating Equations (GEE) model predicting absence of tooth 43 (reference = presence). Coefficients on log scale (*B*, SE, 95% CI) are shown.

| **Fixed effect** | **Estimate** | **SE** | **95%% CI** | ***p*** |
| --- | --- | --- | --- | --- |
| Age: 20–34 | -1.92 | 0.17 | [-2.26, -1.58] | <.001 |
| Age: 60+ | 1.36 | 0.05 | [1.26, 1.46] | <.001 |
| Education attainment: High | -0.79 | 0.08 | [-0.94, -0.63] | <.001 |
| Education attainment: Low | 0.34 | 0.04 | [0.26, 0.41] | <.001 |
| Glycemic status: ≥6.5% | 0.20 | 0.05 | [0.10, 0.31] | <.001 |
| Glycemic status: Low normal <5.4% | -0.07 | 0.06 | [-0.19, 0.05] | .258 |
| Glycemic status: Prediabetes 5.7–6.4% | 0.11 | 0.05 | [0.02, 0.21] | .022 |
| Household income: High | -0.29 | 0.07 | [-0.43, -0.15] | <.001 |
| Household income: Low | 0.45 | 0.04 | [0.36, 0.54] | <.001 |
| NHANES cycle: G | 0.11 | 0.06 | [0.00, 0.22] | .048 |
| NHANES cycle: H | 0.15 | 0.06 | [0.04, 0.26] | .010 |
| NHANES cycle: I | -0.04 | 0.06 | [-0.16, 0.08] | .500 |
| NHANES cycle: J | 0.07 | 0.06 | [-0.04, 0.18] | .199 |
| Sex: Male | -0.02 | 0.04 | [-0.09, 0.06] | .644 |
| Smoking: Ever-smoker | 0.59 | 0.04 | [0.51, 0.67] | <.001 |

**Note**. Intercept terms are suppressed from tooth-level GEE tables for clarity, as they represent the baseline log probability of tooth absence in the reference group and are not clinically interpretable. Reference groups: Female (sex), age 35–59 years (age), medium education, medium household income, never-smoker, high-normal glycaemic status (5.4–5.6% HbA1c), and NHANES cycle F (2009–2010).

## Table S25

Generalized Estimating Equations (GEE) model predicting absence of tooth 44 (reference = presence). Coefficients on log scale (*B*, SE, 95% CI) are shown.

| **Fixed effect** | **Estimate** | **SE** | **95%% CI** | ***p*** |
| --- | --- | --- | --- | --- |
| Age: 20–34 | -0.71 | 0.06 | [-0.83, -0.59] | <.001 |
| Age: 60+ | 0.84 | 0.03 | [0.78, 0.90] | <.001 |
| Education attainment: High | -0.12 | 0.04 | [-0.20, -0.04] | .002 |
| Education attainment: Low | 0.18 | 0.03 | [0.12, 0.23] | <.001 |
| Glycemic status: ≥6.5% | 0.16 | 0.04 | [0.08, 0.24] | <.001 |
| Glycemic status: Low normal <5.4% | 0.09 | 0.04 | [0.01, 0.17] | .028 |
| Glycemic status: Prediabetes 5.7–6.4% | 0.04 | 0.04 | [-0.03, 0.11] | .242 |
| Household income: High | -0.01 | 0.04 | [-0.10, 0.07] | .735 |
| Household income: Low | 0.30 | 0.03 | [0.24, 0.37] | <.001 |
| NHANES cycle: G | 0.03 | 0.04 | [-0.05, 0.12] | .435 |
| NHANES cycle: H | 0.05 | 0.04 | [-0.04, 0.13] | .262 |
| NHANES cycle: I | -0.07 | 0.04 | [-0.15, 0.02] | .120 |
| NHANES cycle: J | -0.01 | 0.04 | [-0.09, 0.07] | .840 |
| Sex: Male | -0.12 | 0.03 | [-0.17, -0.07] | <.001 |
| Smoking: Ever-smoker | 0.34 | 0.03 | [0.29, 0.40] | <.001 |

**Note**. Intercept terms are suppressed from tooth-level GEE tables for clarity, as they represent the baseline log probability of tooth absence in the reference group and are not clinically interpretable. Reference groups: Female (sex), age 35–59 years (age), medium education, medium household income, never-smoker, high-normal glycaemic status (5.4–5.6% HbA1c), and NHANES cycle F (2009–2010).

## Table S26

Generalized Estimating Equations (GEE) model predicting absence of tooth 45 (reference = presence). Coefficients on log scale (*B*, SE, 95% CI) are shown.

| **Fixed effect** | **Estimate** | **SE** | **95%% CI** | ***p*** |
| --- | --- | --- | --- | --- |
| Age: 20–34 | -1.42 | 0.08 | [-1.57, -1.27] | <.001 |
| Age: 60+ | 0.88 | 0.03 | [0.83, 0.94] | <.001 |
| Education attainment: High | -0.53 | 0.04 | [-0.62, -0.45] | <.001 |
| Education attainment: Low | 0.20 | 0.02 | [0.15, 0.24] | <.001 |
| Glycemic status: ≥6.5% | 0.19 | 0.03 | [0.12, 0.26] | <.001 |
| Glycemic status: Low normal <5.4% | -0.03 | 0.04 | [-0.10, 0.04] | .434 |
| Glycemic status: Prediabetes 5.7–6.4% | 0.09 | 0.03 | [0.03, 0.15] | .004 |
| Household income: High | -0.28 | 0.04 | [-0.36, -0.20] | <.001 |
| Household income: Low | 0.25 | 0.03 | [0.20, 0.31] | <.001 |
| NHANES cycle: G | 0.04 | 0.04 | [-0.03, 0.12] | .210 |
| NHANES cycle: H | 0.10 | 0.04 | [0.02, 0.17] | .008 |
| NHANES cycle: I | -0.00 | 0.04 | [-0.07, 0.07] | .921 |
| NHANES cycle: J | 0.06 | 0.04 | [-0.01, 0.13] | .114 |
| Sex: Male | -0.11 | 0.02 | [-0.16, -0.07] | <.001 |
| Smoking: Ever-smoker | 0.42 | 0.02 | [0.37, 0.46] | <.001 |

**Note**. Intercept terms are suppressed from tooth-level GEE tables for clarity, as they represent the baseline log probability of tooth absence in the reference group and are not clinically interpretable. Reference groups: Female (sex), age 35–59 years (age), medium education, medium household income, never-smoker, high-normal glycaemic status (5.4–5.6% HbA1c), and NHANES cycle F (2009–2010).

## Table S27

Generalized Estimating Equations (GEE) model predicting absence of tooth 46 (reference = presence). Coefficients on log scale (*B*, SE, 95% CI) are shown.

| **Fixed effect** | **Estimate** | **SE** | **95%% CI** | ***p*** |
| --- | --- | --- | --- | --- |
| Age: 20–34 | -1.16 | 0.04 | [-1.25, -1.08] | <.001 |
| Age: 60+ | 0.51 | 0.02 | [0.48, 0.54] | <.001 |
| Education attainment: High | -0.49 | 0.03 | [-0.55, -0.44] | <.001 |
| Education attainment: Low | 0.15 | 0.02 | [0.12, 0.18] | <.001 |
| Glycemic status: ≥6.5% | 0.14 | 0.02 | [0.10, 0.18] | <.001 |
| Glycemic status: Low normal <5.4% | -0.09 | 0.02 | [-0.14, -0.04] | <.001 |
| Glycemic status: Prediabetes 5.7–6.4% | 0.07 | 0.02 | [0.04, 0.11] | <.001 |
| Household income: High | -0.24 | 0.03 | [-0.29, -0.19] | <.001 |
| Household income: Low | 0.16 | 0.02 | [0.13, 0.20] | <.001 |
| NHANES cycle: G | 0.03 | 0.02 | [-0.02, 0.07] | .245 |
| NHANES cycle: H | 0.04 | 0.02 | [-0.00, 0.09] | .067 |
| NHANES cycle: I | -0.01 | 0.02 | [-0.06, 0.04] | .707 |
| NHANES cycle: J | 0.02 | 0.02 | [-0.03, 0.06] | .431 |
| Sex: Male | -0.10 | 0.01 | [-0.13, -0.07] | <.001 |
| Smoking: Ever-smoker | 0.26 | 0.02 | [0.23, 0.29] | <.001 |

**Note**. Intercept terms are suppressed from tooth-level GEE tables for clarity, as they represent the baseline log probability of tooth absence in the reference group and are not clinically interpretable. Reference groups: Female (sex), age 35–59 years (age), medium education, medium household income, never-smoker, high-normal glycaemic status (5.4–5.6% HbA1c), and NHANES cycle F (2009–2010).

## Table S28

Generalized Estimating Equations (GEE) model predicting absence of tooth 47 (reference = presence). Coefficients on log scale (*B*, SE, 95% CI) are shown.

| **Fixed effect** | **Estimate** | **SE** | **95%% CI** | ***p*** |
| --- | --- | --- | --- | --- |
| Age: 20–34 | -1.16 | 0.05 | [-1.25, -1.06] | <.001 |
| Age: 60+ | 0.62 | 0.02 | [0.59, 0.66] | <.001 |
| Education attainment: High | -0.51 | 0.03 | [-0.57, -0.45] | <.001 |
| Education attainment: Low | 0.15 | 0.02 | [0.12, 0.18] | <.001 |
| Glycemic status: ≥6.5% | 0.18 | 0.02 | [0.13, 0.23] | <.001 |
| Glycemic status: Low normal <5.4% | -0.10 | 0.03 | [-0.15, -0.05] | <.001 |
| Glycemic status: Prediabetes 5.7–6.4% | 0.09 | 0.02 | [0.04, 0.13] | <.001 |
| Household income: High | -0.25 | 0.03 | [-0.30, -0.19] | <.001 |
| Household income: Low | 0.21 | 0.02 | [0.17, 0.25] | <.001 |
| NHANES cycle: G | -0.00 | 0.03 | [-0.06, 0.05] | .854 |
| NHANES cycle: H | 0.08 | 0.02 | [0.03, 0.13] | .002 |
| NHANES cycle: I | -0.02 | 0.03 | [-0.07, 0.03] | .498 |
| NHANES cycle: J | 0.01 | 0.02 | [-0.04, 0.06] | .703 |
| Sex: Male | -0.04 | 0.02 | [-0.07, -0.00] | .030 |
| Smoking: Ever-smoker | 0.31 | 0.02 | [0.27, 0.34] | <.001 |

**Note**. Intercept terms are suppressed from tooth-level GEE tables for clarity, as they represent the baseline log probability of tooth absence in the reference group and are not clinically interpretable. Reference groups: Female (sex), age 35–59 years (age), medium education, medium household income, never-smoker, high-normal glycaemic status (5.4–5.6% HbA1c), and NHANES cycle F (2009–2010).
